# Transjugular Intrahepatic Portosystemic Shunt for Portal Vein Thrombosis in Cirrhotic Patients: 18-Year Experience in a Tertiary Referral Hospital

**DOI:** 10.3390/diagnostics15222878

**Published:** 2025-11-13

**Authors:** Sara Barranco Acosta, María Sagrario Lombardo Galera, Pedro Blas García Jurado, María Eugenia Pérez Montilla, Antonio Jesús Láinez Ramos-Bossini, Juan José Espejo Herrero

**Affiliations:** 1 Interventional Radiology Unit, Department of Radiology, Hospital Universitario Reina Sofía, 14004 Córdoba, Spain; sarabarrancoacosta@gmail.com (S.B.A.); mslombgalera@hotmail.com (M.S.L.G.); pedroblasgj@gmail.com (P.B.G.J.); marigenperez.montilla@gmail.com (M.E.P.M.); juanjoseespejo@gmail.com (J.J.E.H.); 2 Maimónides Biomedical Research Institute of Córdoba (IMIBIC), 14004 Córdoba, Spain; 3 Advanced Medical Imaging Group, Instituto de Investigación Biosanitaria de Granada (ibs.GRANADA), 18014 Granada, Spain; 4 Department of Human Anatomy and Embryology, School of Medicine, University of Granada, 18016 Granada, Spain

**Keywords:** portal vein thrombosis, transjugular intrahepatic portosystemic shunt, liver cirrhosis, TIPS dysfunction, interventional radiology

## Abstract

**Background**: Transjugular intrahepatic portosystemic shunt (TIPS) has emerged as a feasible therapeutic option for cirrhotic patients with portal vein thrombosis (PVT). This study aimed to assess the long-term outcomes and factors associated with TIPS dysfunction in cirrhotic patients with PVT over an 18-year period in our institution. **Methods**: A retrospective study was conducted at Hospital Universitario Reina Sofía (Córdoba, Spain), including adult and pediatric cirrhotic patients with PVT who underwent TIPS between January 2006 and December 2024. Patient characteristics, procedural techniques, and clinical outcomes were evaluated. The primary outcomes were TIPS insertion success rate, primary patency, and dysfunction (stenosis or occlusion). Bivariate comparisons, logistic regression and receiver-operating characteristic (ROC) analyses were performed to identify potential predictors of TIPS dysfunction. Survival analyses using the Kaplan–Meier method and log-rank test, complemented by Cox regression, were also conducted. **Results**: A total of 36 patients (mean age, 44.8 ± 20.1 years old; 22.2% women; 19.4% children) were included, with a mean follow-up of 66.3 ± 45.9 months and nine deaths (one attributable to the procedure). The primary success rate of TIPS placement was 100%, and mean primary patency was 40.3 ± 40.2 months. TIPS dysfunction occurred in 30.3% of patients. Logistic regression identified age as the only significant predictor of TIPS dysfunction (OR = 0.949; 95%CI, 0.907–0.985, *p* = 0.011). ROC analysis demonstrated an AUC of 0.737 (95%CI, 0.547–0.927), with an optimal age cut-off of 21 (equivalent to 18 years; sensitivity = 91.3%, specificity = 50%). When age was dichotomized into adult versus pediatric groups, the OR was 0.095 (95%CI, 0.011–0.560), consistent with survival analyses (log-rank *p* = 0.007; HR = 4.85; 95%CI 1.36–16.88, *p* = 0.015). **Conclusions**: TIPS is an effective treatment for cirrhotic patients with PVT, achieving high technical success and long-term patency. However, it is not exempt from complications, including death, and potential dysfunction remains a concern, particularly in pediatric patients. Further prospective studies with larger cohorts are warranted to refine patient selection and optimize outcomes.

## 1. Introduction

Portal vein thrombosis (PVT) is a relatively common complication in cirrhotic patients that leads to raised portal vein pressure and reduced hepatic blood flow, worsening liver function [1]. Its prevalence ranges from less than 1% in patients with compensated cirrhosis to 8–25% in candidates for liver transplantation [2]. In addition, incidence rates between 3.5% and 4.6% at 1 year and peak incidences exceeding 10% at 5 years have been reported [3,4].

Anticoagulation with low molecular weight heparins or direct oral anticoagulants—particularly with non-warfarin derived agents (e.g., enoxaparin or rivaroxaban)—is the mainstay of treatment for PVT in cirrhotic patients, aiming to achieve recanalization of the thrombosed vein, prevent thrombus progression, and reduce the risk of complications such as variceal bleeding and worsening hepatic function [5]. Transjugular intrahepatic portosystemic shunt (TIPS) represents an alternative therapeutic approach indicated in specific scenarios, including for treating patients with chronic PVT who do not respond to anticoagulation therapy after 6 months [6] and those presenting with complications of portal hypertension such as refractory ascites [3,7], as well as for secondary prevention following bleeding episodes, particularly of variceal origin [8].

Currently, the American Association for the Study of Liver Diseases (AASLD) recommends considering TIPS in patients with advanced PVT and recurrent bleeding or refractory ascites not manageable by medical or endoscopic means [9]. TIPS can achieve high rates of portal vein recanalization and improve clinical outcomes, including reduced variceal rebleeding and better control of ascites [6,10]. The American College of Radiology (ACR) also acknowledges the technical challenges and variability in outcomes associated with TIPS in patients with PVT, noting that although TIPS is increasingly considered for these patients, the evidence base is still evolving [11].

Previous studies have shown that TIPS implantation in cirrhotic patients with PVT may be highly successful in expert hands. For instance, published meta-analyses showed a high technical success rate (95%,) with pooled rebleeding and 12-month portal vein recanalization rates of 13% and 79%, respectively [8,12]. However, most of the currently available evidence derives from case series with small sample sizes [13,14,15], and factors such as operator experience, procedural technique, approach, PVT location, or age, may play a differentially influence outcomes. Therefore, further research in this area remains warranted.

Moreover, the development of TIPS dysfunction—such as stenosis or loss of patency—is not uncommon, and its severity depends on several factors. Fortunately, in most cases, TIPS dysfunction can be resolved with interventional procedures. For example, stent stenosis can be managed with balloon angioplasty and/or stent dilatation [16]. However, in some cases, TIPS occlusion cannot be restored, leading to recurrence of portal hypertension and potential complications. Hence, improving our understanding of the factors associated with TIPS complications, particularly those enabling early detection and intervention, remains essential.

We previously described our 10-year experience with TIPS implantation in patients with PVT [17]. The aim of this study is to expand and update the series with our current 18-year experience in cirrhotic patients. As a secondary objective, we aimed to identify factors potentially associated with the development of TIPS dysfunction, particularly stenosis and occlusion.

## 2. Materials and Methods

### 2.1. Study Design and Eligibility Criteria

We performed a retrospective observational study at Hospital Universitario Reina Sofía (Córdoba, Spain), a tertiary referral center serving a population of approximately one million inhabitants and equipped with specialized units in interventional radiology and liver transplantation.

**Figure 1:**
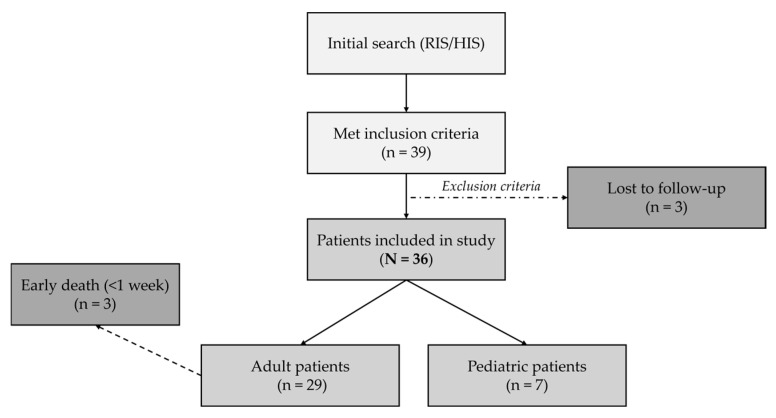
Flowchart of patients included in the study. RIS, radiology information system. HIS, hospital information system.

A systematic search of our radiology and hospital information systems was performed to identify cirrhotic patients with PVT who underwent TIPS placement in our department between June 2006 and June 2024. The inclusion criteria were: (1) structural changes suggestive of liver cirrhosis determined by clinical, imaging and/or histological findings; and (2) PVT confirmed by imaging examinations (Doppler ultrasound, contrast-enhanced CT or MRI). The only exclusion criterion was loss to follow-up within the first 3 months after TIPS placement, but patients who died during this period were included in the analysis. Figure 1 shows the flowchart of patients included in the study.

The Strengthening the Reporting of Observational Studies in Epidemiology (STROBE) guidelines [18] were followed for the design and writing of this study, which was approved by the Provincial Ethics Committee of Córdoba (code, SICEIA-2025-000929). Due to the retrospective nature of the study, written informed consent was waived.

### 2.2. Technical Procedure

Before starting TIPS placement, the portal venous system was imaged using computed tomography (CT) and angiography, including a portal venous phase, to assess the extent and chronicity of the thrombus and to plan the most appropriate approach. All procedures were performed under general anesthesia because patients with PVT are often subject to complex recanalizations that require precision and prolonged procedural times.

TIPS creation was performed following three main therapeutic strategies, depending on different anatomical aspects and PVT chronicity:

(1) Classic transjugular approach (Figure 1)

After ultrasound-guided puncture of the right internal jugular vein, a 10 F introducer was advanced into the right atrium (RA), and a catheter with an angled tip was used to select the right (or occasionally middle) hepatic vein. Transhepatic punctures of the right intrahepatic portal branches were performed using a 16 G Rösch–Uchida needle (Ring-TIPS 200, Cook Inc., Bjaeverskov, Denmark), with or without abdominal ultrasound guidance. In some cases, a Colapinto needle with a 5 F trocar was used as an alternative.

Once the portal branch was accessed, a 0.035″ hydrophilic guidewire was advanced toward the proximal origin of the occlusion. Keeping the guidewire in position, the Rösch–Uchida sheath was exchanged for a 4–5 F vertebral-type catheter to recanalize the occluded segment with a 0.035″ curved hydrophilic guidewire (or alternatively a 0.018″ Astato-30 guidewire, Asahi Intecc, Seto, Japan). After crossing the occlusion—usually straightforward in acute thromboses—, angioplasty was performed using an 8 × 40 mm conventional balloon, followed by deployment of a 10 mm ePTFE-covered stent (Viatorr^®^, WL Gore & Associates, Flagstaff, AZ, USA). The stent was initially dilated to 8 mm and, if the portosystemic (i.e., transhepatic) gradient exceeded 12 mmHg, further dilated to 10 mm to minimize the risk of variceal bleeding.

In most patients (*n* = 31), additional overlapping coaxial stents (self-expandable or balloon-expandable) were required to fully cover the thrombus up to the healthy portal segment. This strategy was common in our series, as complete coverage of the thrombosed segment was considered essential for long-term TIPS patency.

**Figure 2:**
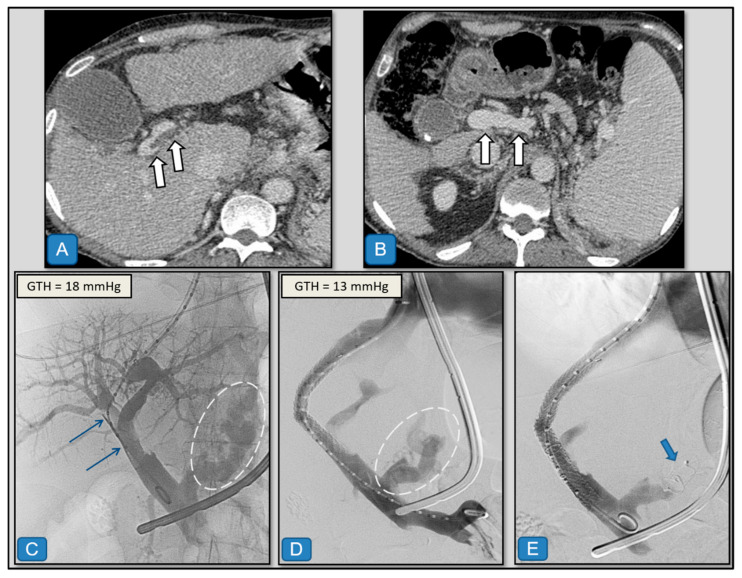
Classic transjugular approach in a 48-year-old man with alcoholic and hepatitis C virus infection cirrhosis with upper gastrointestinal bleeding due to gastroesophageal varices. (**A**,**B**) Contrast-enhanced computed tomography, axial images in portal venous phase. Cirrhotic liver with a partial acute thrombus in the right portal vein (arrows in (**A**)), with patent porta hepatis and splenoportal axis (arrows in (**B**)). (**C**) Using a standard ultrasound-guided access through the right internal jugular vein, puncture was performed into the partially thrombosed right portal branch. Direct portography demonstrates an acute partial thrombosis of the main portal vein that extends to the right portal branch (blue arrows) and opacification of gastroesophageal varices (dashed ovals). The pre-TIPS transhepatic gradient (GTH) was 18 mmHg. (**D**) A 0.035″ hydrophilic guidewire and catheter were advanced through the thrombus, and a TIPS was placed. A 10 mm uncovered stent was deployed, overlapping toward the origin of the superior mesenteric vein, covering the partially thrombosed segment. Both stents were sequentially dilated with conventional 8 and 10 mm balloons, achieving a final post-TIPS GTH of 13 mmHg. Finally, embolization of the left gastric vein was performed with a 14 mm Amplatzer plug (AGA Medical Corporation, Golden Valley, MN, USA) (blue arrow in (**E**)).

**Figure 3:**
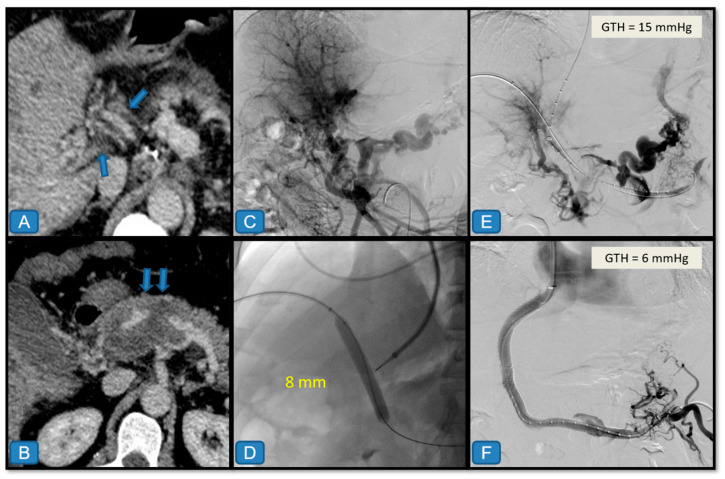
Combined transjugular and transhepatic approach in a 55-year-old man with liver cirrhosis of unknown etiology and bleeding from gastroesophageal varices. (**A**,**B**) Contrast-enhanced computed tomography (portal venous phase) shows chronic portal thrombosis of the main portal vein and splenoportal axis (arrows), with features of cavernomatosis. (**C**) Indirect portography shows a chronic-appearing thrombosis of the portal vein trunk with extensive periportal collateral circulation (cavernomatosis) that recanalizes intrahepatic portal branches. (**D**) Ultrasound-guided percutaneous transhepatic puncture was performed with a 21 G needle to access a right portal branch. A 6 F introducer sheath was then placed, through which a 5 F catheter and a 0.035″ hydrophilic guide were introduced, achieving recanalization of the portal occlusion through angioplasty with a conventional 8 mm balloon. Subsequently, through transjugular access and using the balloon as a target to reach the portal vein from the middle hepatic vein, it was punctured with a 16 G needle to access the portal-splenic system (**E**). The transhepatic gradient (GTH) was 15 mmHg. (**F**) A TIPS was created using an ePTFE-covered stent, with an additional uncoated stent extension into the splenic vein. Both stents were remodeled with an 8 mm balloon, achieving a final GTH of 6 mmHg. Finally, embolization of the transhepatic tract was performed using Gelfoam.

(2) Combined transjugular and percutaneous approach (Figure 2 and Figure 3)

This approach was used when recanalization through the transjugular route alone was not feasible. The procedure began with a standard transjugular access, followed by ultrasound-guided percutaneous puncture of a right intrahepatic portal branch when contrast-enhanced CT or indirect portography demonstrated PVT with patent intrahepatic branches. A 21 G needle (Accustick puncture set, Boston Scientific, Marlborough, MA, USA) was used for the initial puncture, which was then opacified with contrast under fluoroscopic control. The portal branch was catheterized using a 0.018″ guidewire, and the needle exchanged for the set sheath. After confirmation of correct portal positioning, the sheath was replaced by a 6 F introducer (with a 0.035″ semi-rigid guidewire), through which a 4–5 F rigid catheter was advanced to cross the thrombus with a 0.035″ hydrophilic guidewire.

Once recanalization of the portal thrombus was achieved and the splenic hilum or distal superior mesenteric vein (SMV) reached, direct portography was performed with a 5 F pigtail catheter with radiopaque markers. Portal and hepatic vein pressures were recorded to calculate the portosystemic gradient. Conventional balloon angioplasty (8 × 40 mm) was then performed, and a standard TIPS was inserted, often extended with uncovered stents into the splanchnic territory to restore hepatopetal flow.

When puncture of an intrahepatic portal branch was not feasible under ultrasound or fluoroscopic guidance, a target technique was used (e.g., ultrasound-guided implantation of a periportal coil). Alternatively, a balloon (inflated with 50% diluted contrast), snare, or opacified bile ducts were used as fluoroscopic landmarks for targeting. For example, under fluoroscopic control, an 8 × 40 mm balloon placed at the portal bifurcation could be punctured transjugularly with a 16 G Rösch–Uchida needle, using oblique projections for orientation. Successful puncture was confirmed by balloon rupture and contrast extravasation, after which a 0.035″ hydrophilic guidewire was advanced through the broken balloon into the portal system.

When transhepatic access was not feasible or unsuccessful, ultrasound-guided transsplenic access was performed. Using the Accustick set, the splenic parenchyma was punctured to reach an intraparenchymal venous branch, which was then catheterized with a 0.018″ guidewire and exchanged for the sheath. A 6 F introducer and a 4–5 F vertebral catheter were used to traverse the thrombus antegradely after direct portography. Angioplasty was performed with an 8 × 40 mm balloon, followed by standard TIPS creation. To prevent bleeding, the transhepatic (or transsplenic) tract was embolized at the end of the procedure using gelatin sponge, coils, plugs, or cyanoacrylate.

**Figure 4:**
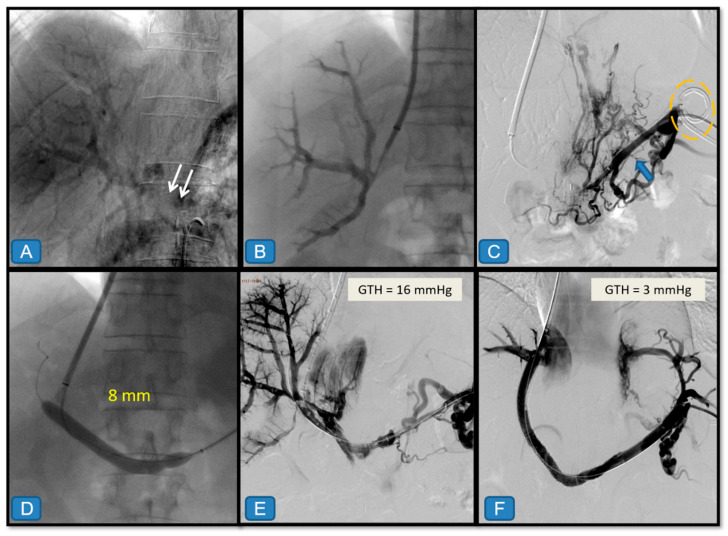
Combined transsplenic approach in a 49-year-old man with history of pancreatic surgery, prehepatic portal hypertension with stenosis of the portomesenteric anastomosis and portal cavernomatosis. (**A**) Indirect portography shows chronic thrombosis of the entire superior mesenteric vein extending through the main portal trunk and the splenomesenteric portal venous confluence (white arrows), with the right and left portal branches remaining patent. (**B**) Through standard transjugular access, direct portography confirmed chronic thrombosis of the entire portal trunk, which could not be successfully recanalized anterogradely despite attempts with various guidewires. (**C**) Under ultrasound guidance, a 10 F catheter was placed in the left flank for ascitic drainage (yellow oval), followed by ultrasound-guided transsplenic puncture (21 G needle) of an intrasplenic venous branch using a 6 F introducer sheath (blue arrow). Direct splenoportography confirms chronic thrombosis of the splenomesenteric portal venous confluence, with opacification of gastroesophageal varices. The transhepatic gradient (GTH) was 16 mmHg. (**D**,**E**) The portal trunk was successfully recanalized retrogradely, predilated with a conventional 8 mm balloon, achieving partial reopening of the vessel. (**F**) A TIPS was created connecting the right hepatic vein with the right portal branch, with an additional 9 mm uncovered stent extending the TIPS toward the patent splenic vein. The final GTH was 3 mmHg. Finally, the transsplenic tract was embolized with cyanoacrylate.

(3) Alternative access through periportal collateral veins (Figure 4)

In cases of chronic PVT with cavernous transformation, direct recanalization of the main portal vein is frequently impossible because the thrombosed segment becomes a fibrous cord. In these patients, TIPS can be created between a hepatic vein and a periportal collateral vessel of sufficient caliber (>3 mm), under combined ultrasound and fluoroscopic guidance. For such purpose, the Rösch–Uchida needle was directed toward the target collateral, typically located at the hepatic hilum, advancing a 0.035″ hydrophilic guidewire through the periportal venous network into the splenic vein or SMV. This strategy allows shunt creation without attempting to reopen the fibrotic portal segment, reducing the risk of liver injury and hemorrhage.

When a suitable periportal collateral could not be identified or accessed, a combined approach was considered, using transjugular and percutaneous routes. In selected cases, a percutaneous mesocaval shunt was created as an alternative, using an anterior abdominal approach under CT or ultrasound guidance. A mesenteric collateral vein was punctured and connected to the inferior vena cava with a retrieval snare or loop to capture the guidewire and establish through-and-through access. This approach could also be performed via mini-laparotomy when percutaneous puncture was technically unfeasible.

This therapeutic option was also successfully applied in pediatric patients with portal cavernomatosis. Despite the smaller caliber of periportal veins, they can still accommodate an 8 mm expanded stent without rupture. The feasibility of this technique depends on the presence of adequate collateral pathways and careful image guidance during the transhepatic puncture phase.

**Figure 5:**
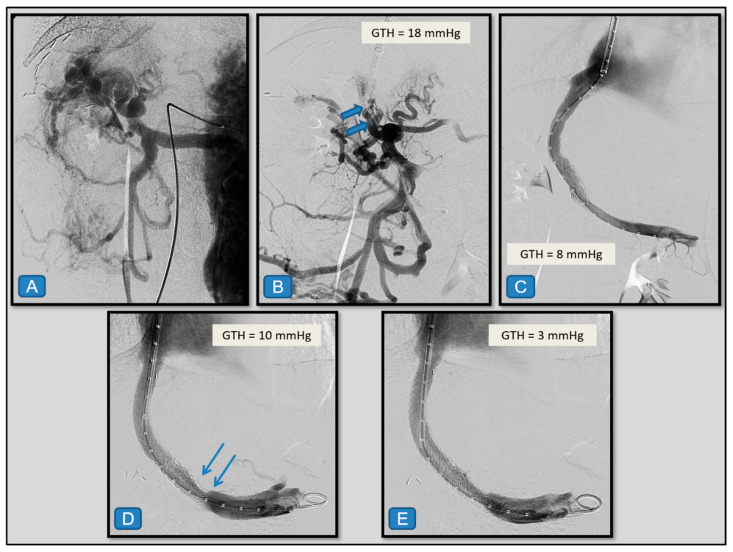
Six-year-old girl with a liver transplant due to extrahepatic biliary atresia, presenting with portal hypertension and bleeding from esophageal varices. (**A**) Indirect portography shows chronic portal thrombosis with cavernomatosis. (**B**) Through the right internal jugular vein, a standard ultrasound-guided access was performed. A 10 F introducer sheath was placed in the right atrium, and an angled catheter was used to select the middle hepatic vein and advance the introducer. From this position, multiple punctures were directed toward a large-caliber collateral vein of the cavernomatosis (blue arrows), yielding a transhepatic gradient (GTH) of 18 mmHg. (**C**) Using a hydrophilic guidewire, this collateral vein was catheterized, and a TIPS was created and dilated with a conventional 8 mm balloon, achieving a final GTH of 8 mmHg. (**D**) Three years after placement, follow-up imaging demonstrated a patent TIPS with stenosis in its distal segment (blue arrows), with a measured gradient of 10 mmHg. (**E**) Angioplasty was performed with an 8 mm balloon through a 7 F introducer, followed by placement of a coaxial 8 mm stent, resulting in a final GTH of 3 mmHg.

Figure 2, Figure 3, Figure 4 and Figure 5 present illustrative examples of TIPS implantation procedures carried out in patients from our series. The cases have been chosen to demonstrate detailed technical aspects of the procedure and corresponding imaging findings in different clinical scenarios. Figure 2 shows a classic transjugular approach in a patient with PVT secondary to liver cirrhosis due to hepatitis C virus infection, where two stents were also deployed and dilated with a balloon. The case depicted in Figure 3 illustrates the usefulness of the combined transjugular-transhepatic approach in a patient with cirrhosis of unknown etiology. In this patient, in addition to TIPS placement, the transhepatic tract was embolized. The case illustrated in Figure 4 was performed using a combined approach in an adult patient with portal vein cavernomatosis. Finally, the case from Figure 5 was a pediatric patient with cavernomatosis and prominent collateral vessels.

### 2.3. Patients Under Study: Definition of Main Variables and Data Collection

Following patient selection according to the eligibility criteria, we collected sociodemographic (age, sex), clinical and procedure-related data, including characteristics of PVT and clinical outcomes. When available, we included variables related to cirrhosis stage such as Model for End-Stage Liver Disease (MELD) scores. For PVT, which was diagnosed by Doppler ultrasound and contrast-enhanced CT, we annotated the type of thrombosis and its location. Relevant variables related to PVT, procedure, and outcomes include the following:

PVT characteristics: Acute thrombosis was defined as a partial or complete filling defect in the portal venous phase, characterized on CT imaging by a non-enhancing intraluminal area within the portal vein, associated with venous enlargement and absence of collateral vessels. Chronic PVT was defined as decreased intraluminal density and reduction in portal vein caliber, often appearing as a fibrotic cord. In chronic cases, cavernous transformation of the portal vein was diagnosed in the presence of multiple periportal collateral veins replacing the normal portal course.

Procedure-related variables: Information was collected on the anatomical access route, portosystemic gradient before and after TIPS (target gradient: ≤12 mmHg), type of stent and balloons used, and stent location relative to relevant anatomical landmarks, including distances from the stent to the RA and to the splenomesenteric portal venous confluence.

Outcome variables: Several outcomes were recorded, including insertion success rate, number of deaths during follow-up (with early death defined as <1 week from the procedure), development of TIPs dysfunction, and primary and secondary patency duration. Technical success was defined as the creation of a TIPS between a hepatic vein and the thrombosed portal vein or large collateral cavernous vessel. TIPS dysfunction was defined as the development of significant stenosis (>50% reduction in vessel diameter relative to the post-TIPS imaging control) or occlusion (absence of contrast opacification) in the presence of clinical symptoms associated with portal hypertension (e.g., variceal bleeding or severe ascites). Confirmation of TIPS dysfunction was obtained by direct portography. TIPS dysfunction diagnosed for the first time in a patient with TIPS permeability was considered as ‘first dysfunction’, while patients with a previous diagnosis of TIPS dysfunction (i.e., repaired on follow-up) were considered to have developed a ‘second dysfunction’. Primary patency was defined as the time from TIPS procedure to first dysfunction or end of follow-up, whereas secondary patency was defined as the duration of TIPS permeability along the follow-up, including cases of first dysfunction that were successfully repaired.

### 2.4. Statistical Analysis

Univariate descriptive analyses were performed for the variables of interest. Qualitative variables were expressed as absolute and relative frequencies, and quantitative variables as mean and standard deviation (SD). The Shapiro–Wilk test was applied to assess the normality of quantitative variables distributions.

To evaluate the secondary objective, bivariate analyses were performed according to the variable “TIPS dysfunction” using chi-square and Student’s t-tests for qualitative and quantitative independent variables, respectively. When the assumptions for these parametric tests were not met, the corresponding non-parametric tests were applied (Fisher’s exact test and Mann–Whitney U test, respectively).

For the variable age, which showed a significant association with TIPS dysfunction, a binary logistic regression was applied to estimate the odds ratio (OR) and 95% confidence interval (CI). Then, its diagnostic performance was assessed by receiver operating characteristic (ROC) analysis, calculating the area under the curve (AUC) with its 95%CI. The optimal cut-off point was determined based on the Youden index.

This threshold was subsequently used to explore the relationship between age and TIPS patency over time using Kaplan–Meier survival curves and the log-rank test. Finally, a Cox proportional hazards model was applied to estimate the hazard ratio (HR) and 95% CI for shunt dysfunction according to the dichotomized age group. No multivariable models were constructed due to the limited sample size.

All analyses were performed with R software version 4.3.2. [19] (Vienna, Austria). Statistical significance was set at *p* < 0.05.

## 3. Results

### 3.1. Characteristics of Patients

A total of 36 patients were included (mean age, 44.8 ± 20.1 years; 22.2% women; 19.4% children). The mean follow-up was 66.3 ± 45.9 months. The etiology of cirrhosis in adults was viral, alcoholic and mixed in 19.4%, 22.2% and 8.3% of cases, respectively. Other etiologies in children included tyrosinemia (n = 1), extrahepatic biliary atresia (n = 1), vascular (n = 3; attributable to porto-sinusoidal vascular disease due to prolonged umbilical vein catheterization in two cases, and to genetic coagulopathy in one case), and idiopathic (n = 2). PVT was chronic in 77.8% of patients, and most of them (58.3% of the total sample) exhibited cavernous transformation. The main indication for TIPS placement was upper gastrointestinal bleeding (94.4%), and the most frequent approach was transjugular (58.3%). The mean portosystemic pressure gradients before and after TIPS placement were 17.3 ± 5.6 mmHg and 8.7 ± 4.1 mmHg, respectively. Target post-TIPS gradient values (≤12 mmHg) were achieved in nearly 80% of patients. None of the patients had an active malignancy at the time of TIPS creation.

Nine patients (25%), including one 10-year-old pediatric patient, died on follow-up. Three deaths (all in adults) occurred within the first week after TIPS placement: two due to multi-organ failure unrelated to the procedure and one due to hemoperitoneum within 48 h following TIPS insertion. The latter occurred in a 68-year-old man with cirrhosis and chronic PVT with cavernous transformation who underwent TIPS due to recurrent upper gastrointestinal bleeding from gastroesophageal varices. The procedure was performed via a transhepatic approach using a single stent and an 8 mm balloon, without collateral or bare stents. During follow-up, TIPS dysfunction occurred in 10 patients (30.3% of cases, excluding early mortality): stenosis in 2 patients (6.1%), occlusion in 6 (18.2%), and concurrent stenosis and occlusion in 2 (6.1%).

Table 1 summarizes the sociodemographic, clinical, procedural, and follow-up characteristics of the study sample.

### 3.2. Factors Associated with the Development of TIPS Dysfunction

Table 2 shows the bivariate comparisons based on the outcome “TIPS dysfunction” during follow-up. The only variable significantly associated with the development of TIPS dysfunction was age, which was significantly higher in patients without dysfunction (*p* = 0.047), in agreement with the findings previously observed between adult and pediatric patients.

### 3.3. Logistic Regression and ROC Analysis for Age as a Predictor of TIPS Dysfunction

Logistic regression analysis including age as the independent variable was statistically significant, with an OR of 0.949 (95%CI, 0.907–0.985; *p* = 0.011), indicating that age acted as a protective factor, with an approximately 5% reduction in the risk of TIPS dysfunction for each additional year of age.

The ROC analysis for age showed an AUC of 0.737 (95%CI, 0.547–0.927). The optimal cut-off value for age according to the Youden index was 21 years, which corresponded to the threshold between pediatric and adult patients (i.e., 18 years), given that no patients were aged between 18 and 20 years old in our sample. At this cutoff, sensitivity and specificity were 91.3% and 50%, respectively (Youden index, 0.423).

**Figure 6:**
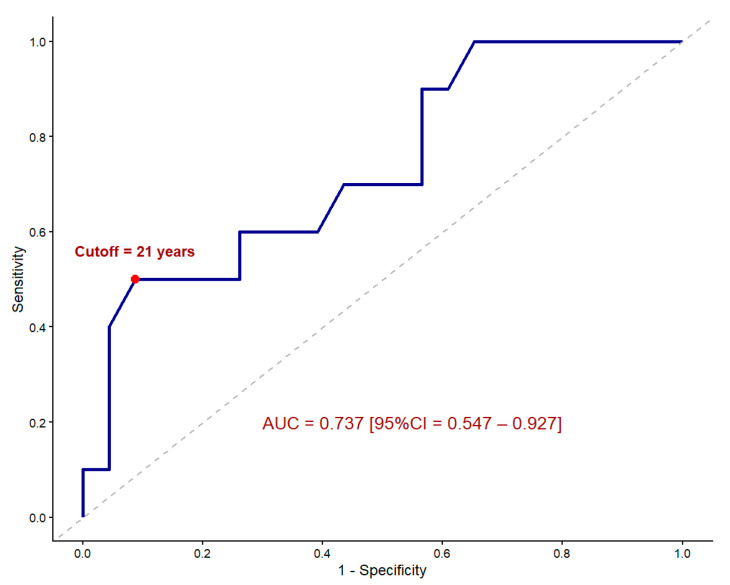
Receiver operating characteristic curve for the prediction of TIPS dysfunction based on age as continuous predictor variable. The optimal cutoff value according to the Youden index was 21 years, equivalent to the threshold between pediatric and adult patients (i.e., 18 years) in our sample. AUC, area under the curve. 95%CI, 95% confidence interval.

When age was analyzed as a dichotomous variable (i.e., adult versus pediatric), the logistic regression model resulted in an OR of 0.095 (95%CI, 0.011–0.576; *p* = 0.016), indicating that adults had nearly a 90% reduction in the risk of developing TIPS dysfunction compared with pediatric patients. Figure 6 shows the corresponding ROC curve.

### 3.4. Kaplan–Meier and Cox Regression Analysis of TIPS Patency According to Age Groups

**Figure 7:**
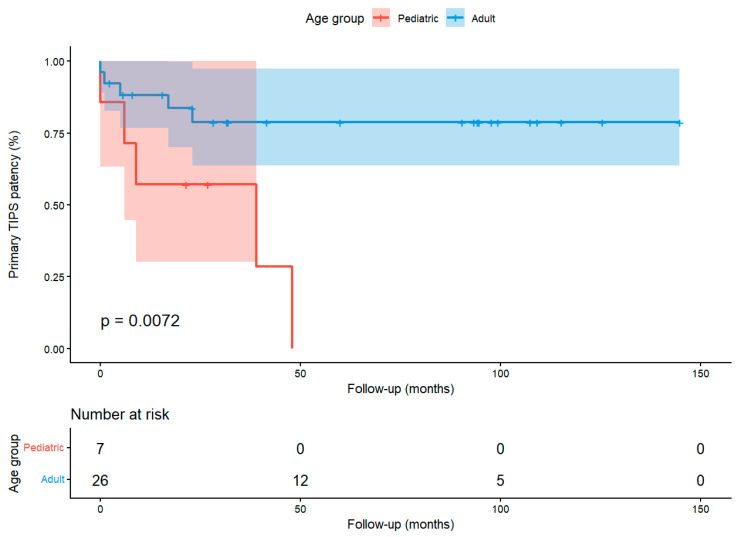
Kaplan–Meier analysis of primary TIPS patency comparing pediatric (<18 years) and adult patients in our sample.

The Kaplan–Meier survival analysis and log-rank test comparing pediatric versus adult patients showed significantly lower primary TIPS patency in the pediatric group (log-rank test, χ^2^ = 7.2, *p* = 0.007). Figure 7 displays the corresponding survival curve.

These results were consistent with the univariate Cox proportional hazards model, which showed that the pediatric group had a significantly higher risk of developing TIPS dysfunction (HR = 4.80, 95%CI, 1.36–16.88, *p* = 0.015).

## 4. Discussion

In this study, we present the results of TIPS placement in cirrhotic patients with PVT treated in a tertiary referral hospital with a dedicated interventional radiology unit over the past 18 years, expanding our previously published series [17]. Our cumulative experience confirms that TIPS leads to high technical (100%) and therapeutic success rates in this population, with a median primary TIPS patency of 40.3 months, and an overall incidence of TIPS dysfunction of approximately one third of cases throughout follow-up. Notably, three early deaths occurred, one of which was directly attributable to the procedure (hemoperitoneum diagnosed within 48 h after TIPS placement). As a secondary objective, we explored factors potentially associated with the development of TIPS dysfunction, and age was the only variable significantly associated with this outcome. Interestingly, clear differences were observed between pediatric (<18 years) and adult patients in both logistic regression and survival analyses (*p* = 0.007), with the former exhibiting a higher risk of TIPS dysfunction in Cox regression (HR = 4.85).

TIPS implantation is widely recognized as a highly effective option for managing PVT in cirrhotic patients, particularly those with refractory thrombosis, symptomatic portal hypertension, or recurrent variceal rebleeding. In refractory PVT, TIPS has shown high recanalization rates. For instance, Mukund et al. (2023) reported complete recanalization in 77.8% of patients and partial recanalization in 16.7%, with significantly better 12-month survival compared to anticoagulation alone [6]. Although our study did not include a control group for direct comparison, our results are consistent with these findings, showing full technical success rate and a median primary stent patency of approximately two years.

The procedure-related mortality in our study (2.8%) aligns with previous reports. Bettinger et al. (2016) reported a 1.5% death rate associated with procedure (0.5%) and shunt-related (1%) failure during hospital stay [20], while Rodrigues et al. (2019) identified only one procedure-related death across 13 studies including 399 patients [12]. Overall, our results support that TIPS remains a relatively safe procedure in experienced hands but calls for further research to identify factors associated with a higher risk of mortality.

The most relevant finding in our series was the clear association between age and TIPS dysfunction. Specifically, five of seven pediatric patients (71.4%) developed TIPS dysfunction, compared with only 19.2% in adults. Previous publications have reported similar findings. For example, Di Giorgio et al. (2020) reported that, in a cohort of pediatric patients with portal hypertension, the primary patency rates of TIPS at 6 months, 1 year, 2 years, and 4 years were 91%, 83%, 60% and 46%, respectively [21]. Similarly, Ghannam et al. (2019) found that TIPS revision was required in 45% of pediatric patients, indicating a significant prevalence of TIPS dysfunction in this population [22]. Additionally, a systematic review and meta-analysis by Hermie et al. reported pooled 2-year primary and secondary patency rates of 61.8% and 99.8%, respectively, in pediatric patients with portal hypertension undergoing TIPS [23].

Several factors may explain the poorer TIPS patency in children. The etiologies of portal hypertension in children (e.g., biliary atresia, tyrosinemia, congenital or porto-sinusoidal vascular disorders) are varied and differ substantially from those in adults [24]. These differences lead to heterogeneous vascular remodeling and a higher prevalence of cavernous transformation and small-caliber vessels, which are less common in adults [25,26]. In addition, other factors such as higher endothelial reactivity or potential mismatch between stent diameter and vascular growth over time could predispose to shunt stenosis or occlusion. Finally, as detailed in Section 2.2, alternative approaches (e.g., transsplenic, periportal collaterals) are more frequent in pediatric cases and might also influence long-term TIPS patency.

Overall, our results support that specific considerations need to be taken into account in children, in agreement with current guidelines that support the need for close surveillance and tailored follow-up in pediatric patients, given the risk of growth-related mismatch and increased endothelial reactivity [27].

Although no other statistically significant differences were found in the explored variables, it is worth discussing some procedure-related findings that could be relevant for future studies. First, patients in whom the post-TIPS portosystemic gradient remained above 12 mmHg showed a higher tendency toward dysfunction, particularly in children, consistent with the concept that suboptimal gradient reduction reflects incomplete decompression of the portal system and may accelerate intimal hyperplasia within the shunt, highlighting the importance of achieving safe portosystemic gradient values after TIPS placement [28,29]. Finally, in the few cases where a covered auxiliary stent was used, TIPS dysfunction was not developed on follow-up. Conversely, most dysfunctions were observed when a bare stent was used. These findings are in agreement with previous literature [30,31] reporting that covered stents are associated with lower rates of TIPS dysfunction compared to bare stents. The use of ePTFE-covered stents in this setting has been shown to improve shunt patency rates significantly.

The major strengths of this study lie in the relatively large sample size with long-term follow-up, considering the low number of patients with PVT treated with TIPS in clinical practice, and the technical details of how the procedure was conducted in different scenarios. However, several limitations should be acknowledged. First, it is retrospective and variables not routinely extracted in our patients could not be analyzed, e.g., elastomeric data (e.g., stiffness of liver and spleen). Some of these variables could be relevant as predictors of outcomes; for instance, inadequate inflow from the SMV or splenic vein or high platelet count have been associated with TIPS dysfunction [32]. Second, its single-centered nature limits the generalizability of our results. Third, the studied sample lacks a control group, which precludes drawing definite conclusions regarding the relative utility of TIPS versus current standard treatment. Finally, procedural expertise is an important determinant of outcomes. Higher operator experience has been consistently associated with improved patency and lower complication rates [30]. Our institution has extensive expertise in TIPS for complex PVT, which likely contributed to the high technical success observed. Nevertheless, caution should be taken when extrapolating these results to centers with less experience. Future studies should address these limitations.

## 5. Conclusions

TIPS represents an effective treatment alternative for cirrhotic patients with PVT. In expert hands, successful insertion is guaranteed, although the procedure is not exempt from potential complications, including, rarely, procedure-related death and, more frequently, TIPS stenosis or occlusion. Most cases of TIPS dysfunction can be successfully managed with interventional revision, ensuring long-term secondary patency. In our cohort, pediatric patients showed a markedly higher risk of TIPS dysfunction, probably due to anatomical and etiological differences compared to adults. Further research is still warranted to validate these results in larger, prospective multicenter studies including both adult and pediatric populations.

## Figures and Tables

**Table 1:** Characteristics of the study sample. Quantitative variables are expressed as mean ± standard deviation (X ± SD) and median (interquartile range). Qualitative variables are expressed as relative (absolute) frequencies [N (%)]. In cases with missing values, data are expressed with respect to the total number of available cases; specifically, three cases of early mortality in adult patients were excluded from non-death related outcomes. * Other causes included: tyrosinemia (n = 1), biliary malformations (n = 2), non-alcoholic fatty liver disease (n = 3), vascular anomalies (e.g., Budd-Chiari, porto-sinusoidal vascular disease) (n = 4), and idiopathic (n = 8). ** Only data for one case is available. *** Two patients (6.1%) developed both stenosis and occlusion. HBV, hepatitis B virus. HCV, hepatitis C virus. PSG, portosystemic gradient. RA, right atrium. SMPVC, splenomesenteric portal venous confluence. SMV, superior mesenteric vein. SV, splenic vein. UGI, upper gastrointestinal bleeding. Significant p-values are highlighted in bold.

Variable	Total Sample (N = 36) X ± SD (Median [IQR]) or N (%)	Adult (n = 29) X ± SD (Median [IQR]) or N (%)	Pediatric (n = 7) X ± SD (Median [IQR]) or N (%)	*p*-Value
Age	44.8 ± 20.1 (50.5 [44.5–57])	53.7 ± 8.9 (54 [48–58])	7.9 ± 4.3 (7 [5–11.5])	**<0.001**
Sex (woman)	8 (22.2)	5 (17.2)	3 (42.9)	0.168
Follow-up (months)	66.3 ± 45.9 (59.8 [26.8–99.2])	64.2 ± 44.7 (56.9 [26.5–98.8])	74.3 ± 53 (65.6 [33–108.6])	0.682
Death on follow-up	9 (25.0)	8 (27.6)	1 (14.3)	0.652
**Cirrhosis etiology**				**0.034**
Alcohol	8 (22.2)	8 (27.6)	0 (0.0)	
Virus (all HCV)	7 (19.4)	7 (24.1)	0 (0.0)	
Alcohol + virus	3 (8.3)	3 (10.3)	0 (0.0)	
Alcohol + HCV	2 (5.6)	2 (6.9)	0 (0.0)	
Alcohol + HBV	1 (2.8)	1 (3.4)	0 (0.0	
Other *	18 (50.0)	11 (37.9)	7 (100.0)	
**TIPS Indication**				1.000
UGI	34 (94.4)	27 (93.1)	7 (100.0)	
Ascites	2 (5.6)	2 (6.9)	0 (0.0)	
**Thrombosis type**				0.309
Acute	8 (22.2)	8 (27.6)	0 (0.0)	
Chronic	28 (77.8)	21 (72.4)	7 (100.0)	
Cavernomatosis	21 (58.3)	14 (48.3)	7 (100.0)	
No cavernomatosis	7 (19.4)	7 (24.1)	0 (0.0)	
**Varicose veins**				1.000
No	4 (11.1)	3 (10.3)	1 (14.3)	
Yes	32 (88.9)	26 (89.7)	6 (85.7)	
Gastroesophageal	26 (72.2)	22 (75.9)	4 (57.1)	
Other	6 (16.7)	4 (13.8)	2 (28.6)	
Pre-TIPS liver transplant	4 (11.1)	2 (6.9)	2 (28.6)	0.163
Post-TIPS liver transplant	4 (11.1)	3 (10.3)	1 (14.3)	1.000
Varicosities embolization	4 (12.1)	4 (15.4)	0 (0.0)	0.555
Pre-TIPS PSG	17.3 ± 5.6 (16 [14–19])	17.9 ± 5.9 (17 [14–19.2])	15 ± 3.2 (15 [13–16.5])	0.247
Post-TIPS PSG	8.7 ± 4.1 (8 [6–12])	9.1 ± 4.2 (8 [6–12.2])	7.0 ± 3.5 (8 [5–8])	0.309
Pre-post TIPS PSG	8.6 ± 5.3 (8 [5–11])	8.8 ± 5.8 (8 [5–11.5])	8.0 ± 2.3 (8 [7–9.5])	0.852
Post-TIPS PSG < 12 mmHg	28 (77.8)	22 (75.9)	6 (85.7)	1.000
**TIPS relative to SMV-SV**				0.078
SV	8 (22.2)	7 (24.1)	1 (14.3)	
SMV	4 (11.1)	1 (3.4)	3 (42.9)	
SMPVC	6 (16.7)	5 (17.2)	1 (14.3)	
≤2 cm from SMPVC	3 (8.3)	2 (6.9)	1 (14.3)	
>2 cm from SMPVC	10 (27.8)	9 (31.0)	1 (14.3)	
Distance to RA (cm)	2.1 ± 1.3 (2 [1–3])	1.9 ± 1.3 (2 [1–3])	2.9 ± 1.1 (3 [2.2–3.8])	0.074
Pre-TIPS MELD	11.4 ± 4 (11 [8–13])	11.2 ± 3.9 (11 [8–12.5])	16 **	0.256
Post-TIPS MELD	12 ± 3.6 (12 [8–14.5])	11.9 ± 3.7 (12 [8–15])	14 **	0.600
**TIPS patency (months)**				
Primary	40.3 ± 40.2 (25 [8.2–57.5])	45.2 ± 43.1 (31 [8.5–90])	21.4 ± 17.8 (21 [7.5–33])	0.296
Secondary	53.4 ± 48 (35.5 [11.2–93.8])	51.9 ± 44.9 (40 [10–93.5])	59.4 ± 62.4 (27 [16.5–103.5])	0.815
TIPS dysfunction ***	10 (30.3)	5 (19.2)	5 (71.4)	**0.016**
Dysfunction (type)				
Stenosis	4 (12.1)	1 (3.8)	3 (42.9)	**0.023**
Occlusion	8 (24.2)	4 (15.4)	4 (57.1)	**0.042**
Dysfunction (time)				
First dysfunction	10 (30.3)	5 (19.2)	5 (71.4)	**0.012**
Second dysfunction	4 (12.1)	3 (11.5)	1 (14.3)	1.000
**Approach**				0.278
Transjugular	21 (58.3)	16 (55.2)	5 (71.4)	
Transhepatic	8 (22.2)	8 (27.6)	0 (0.0)	
Transsplenic	7 (19.4)	5 (17.2)	2 (28.6)	
**Bare stent use**				0.330
No bare stent	5 (13.9)	5 (17.2)	0 (0.0)	
Bare stent 10 mm	23 (63.9)	17 (58.6)	6 (85.7)	
Bare stent 9 mm	5 (13.9)	5 (17.2)	0 (0.0)	
Bare stent 8 mm	3 (8.3)	2 (6.9)	1 (14.3)	
**Viator**				0.566
1 stent	32 (88.9)	25 (86.2)	7 (100.0)	
2 stents	4 (11.1)	4 (13.8)	0 (0.0)	
**Balloon**				0.674
10 mm	15 (41.7)	13 (44.8)	2 (28.6)	
8 mm	21 (58.3)	16 (55.2)	5 (71.4)	
**Target**				0.174
No target	19 (52.8)	16 (55.2)	3 (42.9)	
Balloon	12 (33.3)	10 (34.5)	2 (28.6)	
Bile duct	2 (5.6)	2 (6.9)	0 (0.0)	
Collateral	3 (8.3)	1 (3.4)	2 (28.6)	

**Table 2:** Variables associated with TIPS dysfunction. Quantitative variables are expressed as mean ± standard deviation (X ± SD) and median (interquartile range). Qualitative variables are expressed as relative (absolute) frequencies [N (%)]. In cases with missing values, data are expressed with respect to the total number of available cases; specifically, three cases of early mortality in adult patients were excluded from analyses. HCV, hepatitis C virus. PSG, portosystemic gradient. RA, right atrium. SMPVC, splenomesenteric portal venous confluence. SMV, superior mesenteric vein. SV, splenic vein. UGI, upper gastrointestinal bleeding. Significant p-values are highlighted in bold.

Variable	No TIPS Dysfunction (n = 23) X ± SD or N (%)	TIPS Dysfunction (n = 10) X ± SD or N (%)	*p* Value
Age	49.2 ± 15.9 (51 [46.5–57.5])	29.7 ± 23.6 (30 [7.8–52])	**0.047**
Sex (woman)	5 (21.7)	3 (30.0)	0.673
Follow-up	63.7 ± 44.8 (59.8 [24.8–98.4])	72.3 ± 50.2 (59.8 [40.4–111])	0.603
Death on follow-up	3 (13.0)	3 (30.0)	0.336
**Cirrhosis etiology**			0.316
Alcohol	6 (26.1)	1 (10.0)	
Virus (all HCV)	5 (21.7)	2 (20.0)	
Alcohol + virus	3 (13.0)	0 (0.0)	
Other	9 (39.1)	7 (70.0)	
**TIPS Indication**			1.000
Ascites	2 (8.7)	0 (0.0)	
UGI	21 (91.3)	10 (100.0)	
**Thrombosis type**			0.397
Acute	6 (26.1)	1 (10.0)	
Chronic	17 (73.9)	9 (90.0)	
Cavernomatosis	12 (52.2)	7 (70.0)	
No cavernomatosis	5 (21.7)	2 (20.0)	
**Varicose veins**			1.000
No	3 (13.0)	1 (10.0)	
Yes	20 (87.0)	9 (90.0)	
Gastroesophageal	16 (69.6)	7 (70.0)	
Other	4 (17.4)	2 (20.0)	
Pre-TIPS Liver Transplant	2 (8.7)	2 (20.0)	0.567
Post-TIPS Liver Transplant	3 (13.0)	1 (10.0)	1.000
Varicosities embolization	2 (9.5)	1 (10.0)	1.000
Pre-TIPS PSG	16.5 ± 5.7 (16 [13–18])	18.4 ± 5.6 (16.5 [15–19.5])	0.554
Post-TIPS PSG	8.0 ± 3.7 (7 [6–9.5])	9.7 ± 5 (8 [6.5–13.8])	0.307
Pre-post TIPS PSG	8.5 ± 5.9 (8 [5–10.5])	8.7 ± 3.9 (10 [7–11])	0.554
Post-TIPS PSG < 12 mmHg	20 (87)	6 (60)	0.161
**TIPS relative to SMV-SV**			0.219
SV	4 (20.0)	4 (40.0)	
SMV	2 (10.0)	2 (20.0)	
SMPVC	5 (25.0)	1 (10.0)	
≤2 cm from SMPVC	1 (5.0)	2 (20.0)	
>2 cm from SMPVC	8 (40.0)	1 (10.0)	
Distance to RA (cm)	2.0 ± 1.4 (2 [1–3])	2.3 ± 1.2 (2 [1.6–3])	0.550
Pre-TIPS MELD	11.9 ± 4.2 (11.5 [8.8–12.8])	10.3 ± 3.5 (9 [8–12.2])	0.506
Post-TIPS MELD	12.3 ± 3.5 (12.5 [8.8–15.5])	11.3 ± 4.3 (11 [7.8–13.5])	0.533
**Approach**			0.461
Transjugular	16 (69.6)	5 (50.0)	
Transhepatic	4 (17.4)	2 (20.0)	
Transsplenic	3 (13.0)	3 (30.0)	
**Bare stent use**			0.182
No bare stent	4 (17.4)	0 (0.0)	
10 mm	12 (52.2)	9 (90.0)	
9 mm	4 (17.4)	1 (10.0)	
8 mm	3 (13.0)	0 (0.0)	
**Viator**			1.000
1 stent	20 (87.0)	9 (90.0)	
2 stents	3 (13.0)	1 (10.0)	
**Balloon**			0.257
10 mm	8 (34.8)	6 (60.0)	
8 mm	15 (65.2)	4 (40.0)	
**Target**			0.288
No target	15 (65.2)	4 (40.0)	
Balloon	6 (26.1)	3 (30.0)	
Bile duct	1 (4.3)	1 (10)	
Collateral	1 (4.3)	2 (20)	

## Data Availability

The datasets used and/or analyzed during the current study are available from the corresponding author on reasonable request.
